# A Dose of Defense?: Omega-3 Supplements Appear Protective against PM Effects

**Published:** 2008-09

**Authors:** Cynthia Washam

Omega-3 polyunsaturated fatty acids, believed to lessen the risk of many chronic ailments including arthritis, cancer, heart disease, and memory loss, may also help protect the heart against certain damaging effects of air pollution. In a new study by an international team of researchers, supplementation with omega-3s was associated with significantly reduced cardiac stress caused by particulate matter less than 2.5 μm in diameter (PM_2.5_) in a group of elderly individuals in Mexico City **[*EHP* 116:1237–1242; Romieu et al.]**. The study is the first to examine the effects of omega-3s on biomarkers of cellular response to the oxidative stress of air pollution.

Exposure to high levels of particulates from vehicle exhaust and industrial emissions raises the risk of hypertension, heart arrhythmia, heart attack, and stroke, with the elderly being particularly susceptible. Some of the authors had previously shown both that PM_2.5_ promotes heart disease by diminishing heart-rate variability and that omega-3 supplementation could increase heart-rate variability. The current study was intended to find out how omega-3s achieve their effects.

The study population of 52 elderly nursing home residents was chronically exposed to high PM_2.5_ levels; particulate levels inside the nursing home, where residents spent nearly all their time, correlated with the smoggy surroundings outside. For four months starting in 2001, half the participants in the double-blind study received fish oil supplements at doses typical for over-the-counter supplement users; the other half received soy oil supplements.

The research team compared blood samples taken from subjects before and during supplementation and found that omega-3 use was associated with diminished oxidative damage in blood cells. The observed antioxidant effect of omega-3s was much greater in fish oil users than in soy oil users, a difference the investigators attribute to the different amounts and types of omega-3s in the two supplement types (docosahexaenoic acid and eicosapentaenoic acid in fish oil versus α-linolenic acid in soy oil).

The authors note limitations of their study, such as the small sample size and limited exposure assessment. However, the finding that omega-3s appear effective against oxidative stress related to PM_2.5_ exposure, with fish oil supplements offering more protection than soy oil supplements, merits further study in larger populations.

